# Dynamic Consent: a potential solution to some of the challenges of modern biomedical research

**DOI:** 10.1186/s12910-016-0162-9

**Published:** 2017-01-25

**Authors:** Isabelle Budin-Ljøsne, Harriet J. A. Teare, Jane Kaye, Stephan Beck, Heidi Beate Bentzen, Luciana Caenazzo, Clive Collett, Flavio D’Abramo, Heike Felzmann, Teresa Finlay, Muhammad Kassim Javaid, Erica Jones, Višnja Katić, Amy Simpson, Deborah Mascalzoni

**Affiliations:** 1 Centre for Medical Ethics, Institute of Health and Society, University of Oslo, Blindern, P.O. Box 1130, NO-0318 Oslo, Norway; 2 Norwegian Cancer Genomics Consortium, cancergenomics.no, Oslo, Norway; 3 Centre for Health, Law and Emerging Technologies (HeLEX), Nuffield Department of Population Health, University of Oxford, Oxford, United Kingdom; 4 UCL Cancer Institute, University College London, London, United Kingdom; 5 Norwegian Research Center for Computers and Law, Faculty of Law, University of Oslo, Oslo, Norway; 6 Università degli Studi di Padova, Padova, Italy; 7 Health Research Authority, London, United Kingdom; 8 Focus Area DynAge, Freie Universität, Berlin, Germany; 9 Centre of Bioethical Research & Analysis, NUI Galway, Galway, Ireland; 10 NIHR Musculoskeletal Biomedical Research Unit, Nuffield Department of Orthopaedics, Rheumatology and Musculoskeletal Sciences, University of Oxford, Oxford, United Kingdom; 11 School of Medicine, University of Rijeka, Rijeka, Croatia; 12 Genetic Alliance UK, London, United Kingdom; 13 Centre for Research Ethics and Bioethics, Uppsala University, Uppsala, Sweden; 14 Centre for Biomedicine, EURAC, Bolzano, Italy

**Keywords:** Dynamic consent, Participant engagement, Research communication, Ethics, Biobank, Clinical trials, Clinical research, Software tools

## Abstract

**Background:**

Innovations in technology have contributed to rapid changes in the way that modern biomedical research is carried out. Researchers are increasingly required to endorse adaptive and flexible approaches to accommodate these innovations and comply with ethical, legal and regulatory requirements. This paper explores how Dynamic Consent may provide solutions to address challenges encountered when researchers invite individuals to participate in research and follow them up over time in a continuously changing environment.

**Methods:**

An interdisciplinary workshop jointly organised by the University of Oxford and the COST Action CHIP ME gathered clinicians, researchers, ethicists, lawyers, research participants and patient representatives to discuss experiences of using Dynamic Consent, and how such use may facilitate the conduct of specific research tasks. The data collected during the workshop were analysed using a content analysis approach.

**Results:**

Dynamic Consent can provide practical, sustainable and future-proof solutions to challenges related to participant recruitment, the attainment of informed consent, participant retention and consent management, and may bring economic efficiencies.

**Conclusions:**

Dynamic Consent offers opportunities for ongoing communication between researchers and research participants that can positively impact research. Dynamic Consent supports inter-sector, cross-border approaches and large scale data-sharing. Whilst it is relatively easy to set up and maintain, its implementation will require that researchers re-consider their relationship with research participants and adopt new procedures.

## Background

Conducting biomedical research is essential to increase our understanding of biological and molecular mechanisms underlying disease, test the efficiency of new drugs, interventions and devices, and move toward personalised medicine [1]. Biomedical research requires the continuous collection of biological samples, health and outcome data from representative samples of patients and populations and the follow up of these groups over time [2]. As innovations in technology develop at exponential speed, researchers need to have flexibility in the conduct of their research to be able to react quickly to ongoing developments and accelerate medical discovery and the development of new treatment strategies. However, traditional approaches to the planning and conduct of biomedical research projects present a number of challenges.

First, recruiting enough participants, or reaching out to the population of interest, is often difficult [3, 4]. This is particularly true in genetic research which experiences lower recruitment rates than other types of biomedical research [5]. Participant recruitment may be hindered by a lack of manpower or funding to organise large information-giving and enrolment campaigns. It may also be inhibited by clinical staff’s limited familiarity or understanding of research objectives and fears that it will interrupt patient care [6]. Furthermore, patients may not understand the objectives of the research, may not be willing or able to travel to the research centre [7], or may not speak the language used by researchers [8].

Second, obtaining informed consent from research participants can be demanding. The objective of the informed consent process is to ensure that research participants understand the aims and risks of the research and participate in the research voluntarily with this knowledge. However, research participants often do not understand the content of the information sheet or the consent form for the study, particularly if the consent form is lengthy and includes complex terminology [9, 10]. While some participants may be satisfied with receiving information about the research project only once during the recruitment phase, others may want to go through the information several times and may have additional questions or concerns. Assessing the participants’ health literacy and understanding of the research objectives and implications can be difficult and time-consuming [11]. If new research needs arise that were not foreseen and included in the original consent document, collecting new consent from research participants may be expensive and burdensome, particularly if additional consent requires face-to-face interaction or the mailing of paper consent forms. If multiple consents are collected over time, keeping records of these consents can be complicated, particularly in cohort studies, or in projects spanning several years and multiple iterations where paper consent forms are stored in several institutions [11].

Third, retaining research participants in projects is often challenging [8, 12]. Participants may drop out because they experience changes in their condition, respond adversely to the intervention under investigation, move away from the study area or lose interest in the study. Participant drop-out jeopardises the quality of the data, the significance of research findings, and the conduct of follow-up studies [13]. In addition, clinical researchers often need to collect patient-reported outcome data over time to better understand disease symptoms and the impact on disease burden [14]. Researchers conducting population-based health surveys may want to send new health questionnaires to their participants. Regularly collecting additional data from participants may be administratively demanding and costly, particularly if paper forms have to be sent in the mail.

A number of innovative strategies have been developed which aim to offer practical solutions and tools to facilitate the processes described above. For instance, clinical research projects have developed in-home clinical trial support programs to enable patients who cannot travel long distance to clinical sites to participate in research [12]. Projects are coordinating efforts between research teams and infrastructure support at clinical sites to improve recruitment rates [15]. The use of new technologies such as mobile phones and applications is also explored to facilitate ongoing data collection [16]. In addition, large efforts have been made to investigate designs for informed consent that both facilitate the conduct of biomedical research and protect the interests of research participants. A strategy that has received considerable attention is the use of broad consent. Broad consent is an alternative to the more customary specific consent; many consider specific consent too difficult to apply in biobank research where biological sample collections are built as research resources for multiple uses [17]. Definitions of broad consent vary and span from “consent to a wide (broadly specified) range of options” [18] to consent to “an unspecified range of future research subject to a few content and/or process restrictions” [19]. In general, broad consent can be described as a tool that enables research participants to consent to a variety of research projects. Blanket consent, that is consent to an unlimited range of options, has also been suggested as a potential strategy to facilitate the conduct of research [18]. More recently, meta-consent, an approach which enables individuals to express preferences regarding which type of consent they want to give for which type of research (for example, blanket consent to research on biological samples, specific consent to research led by industrial actors) has been described as a solution that may positively affect people’s willingness to participate in research [20].

In principle, broad and blanket consent may facilitate the conduct of research as researchers do not have to consent research participants each time new research questions or situations arise. However, there is some controversy regarding the extent to which these approaches to consent enable research participants to be truly informed about the objectives and details of the research and are respectful of the participants’ values and personal preferences [18, 21]. Furthermore, it remains unclear whether broad consent may help address some of the challenges described earlier. For instance, it is unlikely that the use of broad consent facilitates reaching out to populations that would not normally participate in research because they do not have easy access to research facilities or do not understand why they should participate. In addition, the use of broad consent may not provide any protection against unforeseen events such as regulatory changes. The difficulties that the Swedish population biobank Lifegene encountered are illustrative of this issue. Although the biobank had already recruited many participants and collected their broad consent, in 2013, the Swedish Data Inspection Board decided to temporarily suspend the biobank’s activities as it considered that broad consent did not describe the biobank research in a way that would satisfy the requirements of forthcoming regulation [22, 23]. Similarly, it is unclear whether meta-consent may positively impact participant retention or facilitate ongoing health data collection in research projects.

Another strategy to facilitate the conduct of biomedical research while protecting the interests of research participants is Dynamic Consent. Dynamic Consent is a term used to describe personalised, online consent and communication platforms [3]. Such platforms are primarily designed to achieve two objectives: 1) facilitate the consent process and 2) facilitate two-way, ongoing communication between researchers and research participants. It should be noted that Dynamic Consent is not the same as specific consent. Rather, it can be setup to accommodate different types of consent depending on the research objectives and context [24]. For instance, biobank research participants may give their broad consent to research through a Dynamic Consent platform. Later in time they may use the platform to give their new consent to new research activities that were not foreseen in the original consent (e.g. feedback of genetic research results), or they may alter their consent choices in response to their changing circumstances [3]. Although a central objective of Dynamic Consent is to offer some flexibility to the consent process, Dynamic Consent platforms may also be used for communication in both clinical and population-based research projects. For instance, researchers may use the platform to give participants regular updates about the research, or ask participants to upload new health data throughout the duration of the research project. Research participants may use the platform to set up their preferences regarding access to their health data by third parties or how often they would like to be contacted by the researchers. In recent years, several clinical and population-based research projects have tested online Dynamic Consent platforms [25–31], initiatives welcomed by research participants [29, 32]. Also in health care settings, projects are exploring the use of online platforms for patient consent to the re-use of electronic patient records for research [33].

In the literature, there is emphasis on discussing how Dynamic Consent may enhance the research participants’ right to make autonomous choices regarding their participation in research, improve their comprehension of the consent process and promote their engagement in the research endeavour [11, 24, 34–37]. Less attention has been given to exploring specific ways that Dynamic Consent may facilitate the conduct of medical research. The key purpose of this paper is to explore how Dynamic Consent can help researchers address the challenges encountered (in population-based and clinical research) when inviting individuals to participate in research and following them up over time in a continuously changing environment.

## Methods

In October 2015, the Centre for Health, Law and Emerging Technologies (HeLEX) [22] at the University of Oxford and Working Group 1 of the COST Action CHIP ME IS1303 “Citizen's Health through public-private Initiatives: Public health, Market and Ethical perspectives” [23] (a European Union Framework which brings together European experts) conducted an interdisciplinary two-day workshop at the University of Oxford to share experiences of implementing Dynamic Consent within research projects. Representatives from a range of disciplines contributed to the workshop including ethicists, lawyers, clinicians, researchers, research nurses and research participants. First, the workshop members discussed Dynamic Consent approaches that are implemented in clinical and biobank research projects and identified key elements in these projects that characterise Dynamic Consent. Several workshop members reported how Dynamic Consent had been designed and applied in their projects. Second, the workshop members mapped a process flowchart describing the main tasks that researchers have to complete in order to include and follow up individuals in research projects. For each task, the workshop members identified challenges encountered that may delay or render task completion difficult. Then a discussion followed as to how Dynamic Consent may facilitate the conduct of each task, particularly in terms of increased requirements for transparency, information-sharing and participant engagement. The workshop members were made aware that the findings from the workshop would be published and were offered the opportunity to contribute to the writing of this paper. Notes were taken during the workshop; the workshop organisers summarised and analysed the data using a content analysis approach with an inductive approach [38]. First, the notes were sorted and the data categorised according to the main tasks that had been listed in the process flowchart. Then the data were condensed to reflect the main points made by the workshop members. A preliminary paper summarising the workshop findings was shared with workshop members for validation. Their comments and corrections were integrated into the results. This research did not require informed consent or approval from an ethics board.

## Results

**Fig. 1:**
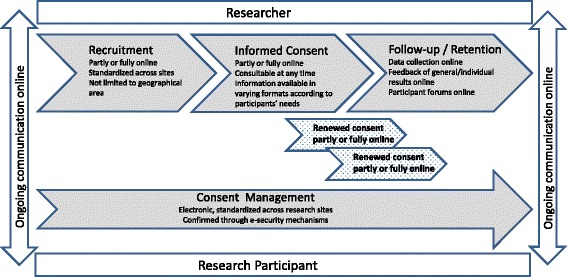
Dynamic Consent’s contribution to phases of research

Discussions at the workshop demonstrated that Dynamic Consent can provide practical solutions to the conduct of four main tasks: participant recruitment, collection of informed consent, participant retention and consent management as summarised below and described in Fig. 1. For each task, we provide concrete examples from research projects to describe how Dynamic Consent can address these challenges.

### Participant recruitment

A Dynamic Consent platform can be set up to provide most of the information about a research project online in a user-friendly and standardised way across research sites (including video, podcast, web, or mobile applications). The information can be given in as many languages as necessary by use of subtitles, dubbed soundtracks and translated text, and at a level of detail that satisfies the specific needs of participants [16, 18]. In principle, the information is accessible to large groups regardless of their geographical location since it is on the Internet, thus potentially broadening the research population. This may be particularly useful in rare disease research where the number of cases is often low and patients are geographically scattered. An example of this is the website for RUDY, a study in rare diseases of the bones, joints and blood vessels headed by a research team at the University of Oxford [39]. The website provides simple information about the research and explains how patients diagnosed with a relevant rare disease, or their relatives, may register to become a research participant in the study. Individuals who would like to register are invited to use the online secure registration system and are informed that they will be contacted after registration for completion of the consent process, usually by telephone, by a research team member [27]. Patients can consult information sheets and consent forms on the website. Since most of the information about the project is available online, the time needed for individual discussions with potential research participants is reduced.

To reach out to populations who are not entirely familiar with the use of online technologies or do not have access to them, the use of a Dynamic Consent platform may be accompanied with conventional methods such as paper leaflets or local information-giving meetings. This strategy was used in the Cooperative Health Research In South Tyrol (CHRIS) study [40], a population-based study aiming to investigate the genetic and molecular basis of age-related common chronic conditions and their interaction with life-style and environment in the general population [28]. To recruit participants from rural areas to the study, village information meetings, broadcasts of information to the public through the local media and web-based recruitment were combined to maximise family participation. However, it is anticipated that the use of multiple methods may become less important as people’s access and familiarity with the Internet improves and they become acquainted with Dynamic Consent platforms. Once participants are recruited, the platform may also be used to regularly update them about recruitment progress and how they can improve awareness of the research within their communities.

### Collection of informed consent

Although standard ethical practice is to have face-to-face dialogue with potential research participants at least for the attainment of the original consent, Dynamic Consent may challenge such practice. In the RUDY study, potential participants discuss the study with the research team in telephone consultations. Then, the participants may choose to download the informed consent form from the website, sign it and send it to the research team by postal mail or email, or they may give their electronic sign-off [27]. Research projects may use mechanisms to assess the participants’ level of comprehension of the research online, for instance by asking research participants to correctly answer interactive questions before providing their consent [33]. As an illustration, potential participants in the Harvard Personal Genome Project (PGP) [41], a project aiming to publicly share the genome, health and trait data of thousands of volunteers, are required to complete an online enrolment examination to demonstrate their comprehension of the risks and protocols associated with being a member of the project before they can give their informed consent to participate in the research [42]. Individuals who fail the examination or do not complete it are not eligible to participate in the research.

A Dynamic Consent platform enables researchers to refine consent in a more nuanced way that the traditional ‘all or nothing’ approach (either participate in the research or not) [43]. For instance, researchers may give participants the choice to consent to some or all aspects of the research depending on their personal preferences and beliefs. Researchers may also ask research participants which types of data access by third parties are acceptable to them. For example, in the Platform for Engaging Everyone Responsibly (PEER) [44], a tool developed by the Genetic Alliance [45] and Private Access [46], research participants may indicate the type of access that they approve and do not approve of, in a matrix. Researchers may also ask participants to consent to a range of other activities at any time; these include data deposit in public research databases [47], data sharing with drug companies and privately-funded institutions [48, 49], or the use of biological samples in case of death or incapacity. This solution enables researchers to know exactly which levels of privacy risks research participants are willing to take and which data may or may not be used in the research. Access preferences can be updated at any time depending on the specific needs and preferences of each research participant and may be directly connected to the databases [50]. Rather than creating potential restrictions on data sharing, such a strategy may enhance data sharing. This is because research participants often consider that the altruistic benefits of sharing their data for research outweigh potential risks [33, 51, 52] but anticipate being consulted about how their data are used and expect transparency regarding such decisions [51, 53].

A Dynamic Consent platform may also enable participants to consent online to the feedback of genetic research results, even years after a project starts. For instance, participants in the CHRIS study may indicate in their personal settings whether they would like to be contacted if results are produced in the research which may offer “chances of therapy or prevention”, and by whom (for example the hospital which recruited them in the research or their general practitioner) [50]. Enabling participants to consent to the feedback of their genetic research results may be useful in research projects planning to recruit participants in future follow-up studies on the basis of their genotype [54]. In principle, the results could be fed back through the platform if the necessary requirements in terms of genetic counselling are met. As an illustration, private actors such as the direct-to-consumer genetic testing company 23andMe combine user-friendly online feedback services with genetic counselling by telephone to those customers who want it [55].

### Participant retention

Dynamic Consent enables researchers to provide participants with regular updates about early findings, follow-up studies, key outcomes, presentations and publications, or invite them to information meetings or follow-up consultations. A recent survey conducted among participants in the CHRIS study shows that ongoing communication motivates research participants to continue with the research [50], an observation also made in previous studies [29, 32]. Importantly, researchers may use a Dynamic Consent platform to ask participants to upload additional health data online. This may become a critical feature of Dynamic Consent as researchers are increasingly moving away from traditional, large-scale randomised controlled trials under which measurements from thousands of people are harvested, to trials conducted on smaller patient groups that are stratified based on presence or absence of specific biomarkers [56]. In these trials, relying solely on statistical methods or average responses to assess new treatment strategies is hardly optimal since the numbers are too small to quantify positive and negative uncertainties such as benefit and risk [57]. Researchers need to rely more on patients’ experiences by regularly collecting data from them regarding health outcomes, quality of life, side effects and personal utility of interventions over time [43]. In the RUDY study, participants use the online platform to fill in health questionnaires, manage their history of illness as well as diagnostics, prescriptions and surgical interventions. The information uploaded in the platform is not only useful to researchers but also to individual research participants who can use it, for instance, in their consultations with physicians [27].

The Dynamic Consent platform may be designed to enable participants to initiate communication with researchers or among themselves, for example via online discussions, forums and webinars. For instance they may do so to comment on recruitment and assessment processes or to identify new research questions that may be relevant to them, thus helping researchers to identify future research opportunities [58]. The use of the platform may enhance participants’ understanding of research and positively impact their willingness to remain in a research project as their scientific literacy increases, as illustrated by low withdrawal rates in the Harvard Genome Project [42] and the CHRIS study (Mascalzoni D, De Grandi A, Pattaro C, D'Elia J, Pramstaller P, Goegele M, et al. Dynamic Consent: building trust through ELSI improvements. Six years of Dynamic Consent in the CHRIS study. Submitted). Participants may also use the platform to indicate when, how, and by what means (e.g. telephone, letter, e-mail, SMS, website) they wish to be notified about new events and in what circumstances, thus mitigating concerns that they may feel overwhelmed by continual communication, or conversely feel insufficiently informed [59].

### Consent management

The Dynamic Consent platform allows consent records to be stored and updated electronically, providing researchers with a reliable and fully tracked overview. Research participants may consult their current and previous consent decisions at any time. The electronic storage of consent details enables researchers to confirm easily the consent status of participants and to audit and review procedures without the need to refer to individual paper records. This can significantly reduce the time taken for audit and improve reliability of the record trail. The technology to gather and securely store Dynamic Consent records can be developed so that it is durable despite rapid software advances and meets security expectations similar to requirements for electronic health records [60]. For instance, online consent may be valid only if provided with an official e-identity or unique user name and password given by public services [30]. In principle, the Dynamic Consent platform can also be linked to other information systems such as Laboratory Information Management Systems in biobanks [24] and electronic patient records [33]. These systems can be automatically updated with the consent preferences of research participants as these are electronically “tagged” to each biological sample and/or data [50]. Participants can enter the platform at any time to review their consents, print a copy, or ask for a copy to be sent by electronic mail or post [31].

## Discussion

The use of Dynamic Consent may facilitate the conduct of specific research tasks, positively impact recruitment and retention and simplify the collection and management of participants’ consent. It may also potentially contribute to reducing costs as many activities (such as sending out information to research participants in the mail) are conducted online, permit greater operability across different organisations if built using recognised national standards [11] and provide researchers with a practical tool to meet forthcoming legal requirements for transparency and precise information-giving to participants [61]. Importantly, Dynamic Consent may offer an opportunity for researchers and research participants to engage in long-term dialogue in a way that goes beyond the traditional one-off consent process, which may benefit both parties. Our results are supported by recent recommendations to explore online tools and multimedia interventions as these have the potential to improve consent processes and increase participants’ level of comprehension of the research [62, 63]. Establishing a culture of ongoing communication between researchers and research participants is increasingly demanded by patient advocacy groups [64] and research participants [53] who would like to be consulted about third party access to their data [51, 65] and the management of genetic research results [66]. Increased researcher-research participant communication is also encouraged by research funders and policymakers to improve the usefulness and accuracy of research, facilitate the development of research projects within areas of medicine that have been given little priority (such as rare diseases) and accelerate the translation of research findings to clinical practice [1, 67]. As an illustration, the recently launched Precision Medicine Initiative Cohort Program [68] which aims to build a national cohort of one million participants across the United States, recommends the development of an online platform for “dynamic information sharing” that enables participants to “actively engage in an informed, voluntary, and ongoing manner”, including setting consent preferences or consulting information whenever needed, and providing “requested data when it is convenient for them” [69].

Dynamic Consent platforms are still under development and only limited empirical data report experiences from their use or hurdles to their implementation [27, 29, 31, 32]. However, several elements need to be considered by researchers before implementing Dynamic Consent platforms in their project.

### Cost and maintenance

Designing, implementing and maintaining a Dynamic Consent platform requires staff with good communication and IT-skills and, in some cases, equipment if potential participants cannot use their own device [16]. However, such costs can reasonably be expected to decrease in the future as online solutions are likely to become more standardised, user-friendly and automatic. If researchers are able to use existing software or are provided with access to a national platform for Dynamic Consent, as was recently proposed in Norway [11], this may also reduce investments in IT-infrastructure [24]. Similarly, links can be provided in the platform to publicly available tools such as educational videos, thus limiting the need to produce information material. Researchers should identify key individuals who may help them develop a Dynamic Consent platform, investigate collaborations with other projects to facilitate platform sharing or establish whether there is a national platform that could be used. A budget for the development or use of a platform should be included in research funding applications.

### Collaboration with research ethics committees

Research ethics committees may not be familiar with Dynamic Consent solutions, although this is gradually changing [70]. The implementation of Dynamic Consent in research projects requires research ethics committees assessing research projects to adapt from expectations of participants’ binary yes/no consent status to one that varies both in scope and across time. Research ethics committees may need to put greater emphasis on evaluating the functionalities of the tools that are made available to participants in a research project rather than scrutinising the type of consent they may give. In the CHRIS study, the research ethics committee approved the whole process of Dynamic Consent along with the informational material. A presentation of the concept had been provided a few weeks earlier. The committee agreed that once a participant was informed about the procedure and gave initial consent in person, he or she could deal with subsequent consent requests online (provided security checks and identification means were in place) [50]. As the use of Dynamic Consent develops, researchers could collaborate with research ethics committees to agree upon required criteria for a platform to qualify as an approved Dynamic Consent platform.

### Accessibility of the platform

Some research participants may not have access to the Internet, may not be the owner of a mobile device, or may not have the ability to use these technologies [33]. This may be particularly true for the elderly, the disabled and individuals in socially deprived communities. Recent experiences from the use of online platforms for consent show that populations enrolling electronically are less diverse in terms of ethnicity and education than populations enrolled through other means [31]. The positive effects of using multimedia platforms in the informed consent process also remain unclear for socio-economically disadvantaged groups [71]. Furthermore, it is unknown whether the use of a Dynamic Consent platform fully addresses participants’ needs in terms of human interaction [32]. Researchers should ensure that education in the use of online tools is provided to participants who request it and that alternative solutions including face-to-face meetings are available for those who cannot use the platform or are not likely to benefit from its use. Younger people are often experts in the use of online communication tools; their input to the design of a Dynamic Consent platform may therefore be useful in addition to contributions from older people who would be potential users.

It is inevitable that some research participants will not want to use the Dynamic Consent platform even if it is user-friendly and easily accessible. As an illustration, some patients who are recruited to research through their clinician may fully trust the clinician’s judgment and prefer to give a one-off broad consent to participation in research considering a Dynamic Consent interface unnecessary. Thus it is important to note that the objective of using a Dynamic Consent platform is not to force people to engage in research and communicate with researchers, but rather to offer them an opportunity to do so. Results from a recent empirical study aiming to investigate biobank participants’ experiences of the use of a Web 2.0 Dynamic Consent interface suggest that once introduced to using an online platform, participants recognise its benefits and find the functions offered by the platform useful [32]. Disseminating information about the platform and enabling participants to discuss the platform together, may indirectly encourage some reluctant participants to consider its use.

### Personal responsibility

Dynamic Consent respects differences between research participants. Researchers must ensure that this empowering feature of the platform does not lead to undesirable side effects [59]. Research participants should not have to take responsibility for making decisions regarding complex issues that they do not fully grasp or are not in a position to assess properly [59, 72]. For instance, as data sharing spans several years and a variety of projects, research participants may struggle to decide which data sharing scenarios are or are not acceptable to them. Researchers may use the platform to educate research participants in the general implications of research including the benefits or risks of data sharing, using short information videos or frequently asked questions. Research participants who have a better understanding of what is at stake are more likely to be able to make informed decisions about data sharing. Finally, participation in genetic and genomic research may have implications not only for the individuals recruited to the project but also for their family. In the future, researchers may need to take this into consideration when designing communication interfaces for participants [73].

## Conclusion

Dynamic Consent, through transparent information exchange and ongoing consent, aims to reinforce the informational, societal and relational value of research and implies a powerful change in the participant’s role from passive ‘subject’ to active ‘participant’ [74]. With Dynamic Consent, informed consent is not restricted to a functional or legal instrument, but also becomes a social agreement between researchers and research participants [75]. Such change is essential to the creation of new knowledge, the development of new and adaptive research designs, and the realisation of personalised medicine [1]. It may however generate some anxiety in the research community as the active role of participants is sometimes perceived as potentially threatening [36]. Similar concerns were raised when the Harvard Personal Genome Project (PGP) implemented an online platform for Open Consent in 2005 [41]. Open consent means that participants give their consent to unrestricted disclosure of their genotype-phenotype data and are made fully aware of the risks of participation in the project, including loss of confidentiality and privacy through public disclosure or identification [76]. In the project, ongoing communication is maintained with participants to collect knowledge regarding the consequences of participation. Experiences from the first ten years of the project show that ongoing communication is perceived as meaningful both by the research participants and the researchers [42].

When engaging participants in research through a Dynamic Consent platform, researchers need to be open-minded, accept that research participants may raise critical questions and make suggestions, and be prepared to take these into consideration in the design of the research. In return, researchers will benefit from having more engaged, committed and productive participants in their research; such participants are useful as demonstrated by the successful contributions to research made by online communities of patients [77]. Dynamic Consent could provide a tool that enables researchers to fully benefit from increased interaction with participants.

## Abbreviations

CHIP ME: Citizen’s health through public-private initiatives: public health, market and ethical perspectives
CHRIS: Cooperative health research in south tyrol
COST Action: European cooperation in science and technology
PEER: Platform for engaging everyone responsibly
PGP: Harvard personal genome project

## References

[1] European Science Foundation. ESF Forward Look: Personalised Medicine for the European Citizen. http://archives.esf.org/fileadmin/Public_documents/Publications/Personalised_Medicine.pdf. Accessed 25 Nov 2016.

[2] Anderson N, Bragg C, Hartzler A, Edwards K (2012). Participant-Centric Initiatives: Tools to Facilitate Engagement In Research. Appl Transl Genom.

[3] Fletcher B, Gheorghe A, Moore D, Wilson S, Damery S (2012). Improving the recruitment activity of clinicians in randomised controlled trials: a systematic review. BMJ Open.

[4] Johnsson L, Helgesson G, Rafnar T, Halldorsdottir I, Chia KS, Eriksson S (2010). Hypothetical and factual willingness to participate in biobank research. Eur J Hum Genet.

[5] Matsui K, Kita Y, Ueshima H (2005). Informed consent, participation in, and withdrawal from a population based cohort study involving genetic analysis. J Med Ethics.

[6] Ross S, Grant A, Counsell C, Gillespie W, Russell I, Prescott R (1999). Barriers to participation in randomised controlled trials: a systematic review. J Clin Epidemiol.

[7] Newington L, Metcalfe A (2014). Factors influencing recruitment to research: qualitative study of the experiences and perceptions of research teams. BMC Med Res Methodol.

[8] Boden-Albala B, Carman H, Southwick L, Parikh NS, Roberts E, Waddy S (2015). Examining Barriers and Practices to Recruitment and Retention in Stroke Clinical Trials. Stroke.

[9] Perry J, Wohlke S, Hessling AC, Schicktanz S. Why take part in personalised cancer research? Patients' genetic misconception, genetic responsibility and incomprehension of stratification-an empirical-ethical examination. Eur J Cancer Care (Engl) 2016. 10.1111/ecc.12563 27507437

[10] D'Abramo F, Schildmann J, Vollmann J (2015). Research participants' perceptions and views on consent for biobank research: a review of empirical data and ethical analysis. BMC Med Ethics.

[11] Budin-Ljosne I, Bentzen HB, Solbakk JH, Myklebost O (2015). Genome sequencing in research requires a new approach to consent. Tidsskr Nor Laegeforen.

[12] Khaleel SL. In: Clinical Leader. Rare Disease Patient Recruitment And Retention. http://www.clinicalleader.com/doc/rare-disease-patient-recruitment-and-retention-0001. Accessed 25 Nov 2016.

[13] Penckofer S, Byrn M, Mumby P, Ferrans CE (2011). Improving subject recruitment, retention, and participation in research through Peplau's theory of interpersonal relations. Nurs Sci Q.

[14] Bronstein MG, Kakkis ED (2016). Patients as key partners in rare disease drug development. Nat Rev Drug Discov.

[15] Nicholson LM, Schwirian PM, Klein EG, Skybo T, Murray-Johnson L, Eneli I (2011). Recruitment and retention strategies in longitudinal clinical studies with low-income populations. Contemp Clin Trials.

[16] Pugliese L, Woodriff M, Crowley O, Lam V, Sohn J, Bradley S (2016). Feasibility of the "Bring Your Own Device" Model in Clinical Research: Results from a Randomized Controlled Pilot Study of a Mobile Patient Engagement Tool. Cureus.

[17] Hansson MG, Dillner J, Bartram CR, Carlson JA, Helgesson G (2006). Should donors be allowed to give broad consent to future biobank research?. Lancet Oncol.

[18] Hofmann B (2009). Broadening consent--and diluting ethics?. J Med Ethics.

[19] Grady C, Eckstein L, Berkman B, Brock D, Cook-Deegan R, Fullerton SM (2015). Broad Consent for Research With Biological Samples: Workshop Conclusions. Am J Bioeth.

[20] Ploug T, Holm S (2015). Going Beyond the False Dichotomy of Broad or Specific Consent: A Meta-Perspective on Participant Choice in Research Using Human Tissue. Am J Bioeth.

[21] Caulfield T, Upshur RE, Daar A (2003). DNA databanks and consent: a suggested policy option involving an authorization model. BMC Med Ethics.

[22] Lind A-S. In: Uppsala Universitet. New law for Biobank researchers http://www.crb.uu.se/biobank-perspectives/item/?tarContentId=496836. Accessed 25 Nov 2016.

[23] Lind A-S. LifeGene - a Closed Case? In: Information and Law in Transition: Freedom of Speech, the Internet, Privacy and Democracy in the 21st Century. Edited by Lind AS RJ, Österdahl I. Stockholm: Liber; 2015. p. 339–50.

[24] Kaye J, Whitley EA, Lund D, Morrison M, Teare H, Melham K (2015). Dynamic consent: a patient interface for twenty-first century research networks. Eur J Hum Genet.

[25] Wilbanks J, Friend SH (2016). First, design for data sharing. Nat Biotechnol.

[26] Dixon WG, Spencer K, Williams H, Sanders C, Lund D, Whitley EA (2014). A dynamic model of patient consent to sharing of medical record data. BMJ.

[27] Javaid MK, Forestier-Zhang L, Watts L, Turner A, Ponte C, Teare H (2016). The RUDY study platform - a novel approach to patient driven research in rare musculoskeletal diseases. Orphanet J Rare Dis.

[28] Pattaro C, Gogele M, Mascalzoni D, Melotti R, Schwienbacher C, De Grandi A (2015). The Cooperative Health Research in South Tyrol (CHRIS) study: rationale, objectives, and preliminary results. J Transl Med.

[29] Teare HJ, Morrison M, Whitley EA, Kaye J. Towards ‘Engagement 2.0’: Insights from a study of dynamic consent with biobank participants. Digital Health. 2015;0(0):1–13. 10.1177/2055207615605644 PMC6001239 29942545

[30] Thiel DB, Platt J, Platt T, King SB, Fisher N, Shelton R (2015). Testing an online, dynamic consent portal for large population biobank research. Public Health Genomics.

[31] Boutin NT, Mathieu K, Hoffnagle AG, Allen NL, Castro VM, Morash M, et al. Implementation of Electronic Consent at a Biobank: An Opportunity for Precision Medicine Research. J Pers Med. 2016;6(2):17. 10.3390/jpm6020017 PMC4932464 27294961

[32] Coathup V, Teare HJ, Minari J, Yoshizawa G, Kaye J, Takahashi MP (2016). Using digital technologies to engage with medical research: views of myotonic dystrophy patients in Japan. BMC Med Ethics.

[33] Spencer K, Sanders C, Whitley EA, Lund D, Kaye J, Dixon WG (2016). Patient Perspectives on Sharing Anonymized Personal Health Data Using a Digital System for Dynamic Consent and Research Feedback: A Qualitative Study. J Med Internet Res.

[34] Kaye J, Curren L, Anderson N, Edwards K, Fullerton SM, Kanellopoulou N (2012). From patients to partners: participant-centric initiatives in biomedical research. Nat Rev Genet.

[35] Cañada JA, Tupasela A, Snell K (2015). Beyond and within public engagement: a broadened approach to engagement in biobanking. New Genet Soc.

[36] D'Abramo F (2015). Biobank research, informed consent and society. Towards a new alliance?. J Epidemiol Community Health.

[37] Williams H, Spencer K, Sanders C, Lund D, Whitley EA, Kaye J (2015). Dynamic consent: a possible solution to improve patient confidence and trust in how electronic patient records are used in medical research. IMIR Med Inform.

[38] Elo S, Kyngas H (2008). The qualitative content analysis process. J Adv Nurs.

[39] The Rudy Study. https://research.ndorms.ox.ac.uk/rudy/. Accessed 25 Nov 2016.

[40] The CHRIS Study (Cooperative Health Research In South Tyrol). In: EURAC Research. http://www.eurac.edu/en/research/health/biomed/projects/Pages/default.aspx. Accssed 25 Nov 2016.

[41] Harvard Personal Genome Project. http://www.personalgenomes.org/. Accessed 25 Nov 2016.

[42] Ball MP, Bobe JR, Chou MF, Clegg T, Estep PW, Lunshof JE (2014). Harvard Personal Genome Project: lessons from participatory public research. Genome Med.

[43] Melham K, Moraia LB, Mitchell C, Morrison M, Teare H, Kaye J (2014). The evolution of withdrawal: negotiating research relationships in biobanking. Life Sci Soc Policy.

[44] Platform for Engaging Everyone Responsibly (PEER). In: Genetic Alliance. http://www.geneticalliance.org/programs/biotrust/peer. Accessed 25 Nov 2016.

[45] Genetic Alliance. http://www.geneticalliance.org/. Accessed 25 Nov 2016.

[46] Private Access, Inc. https://www.privateaccess.info/. Accessed 25 Nov 2016.

[47] Haga SB, O'Daniel J (2011). Public perspectives regarding data-sharing practices in genomics research. Public Health Genomics.

[48] Nilstun T, Hermeren G (2006). Human tissue samples and ethics--attitudes of the general public in Sweden to biobank research. Med Health Care Philos.

[49] Critchley C, Nicol D, Otlowski M (2015). The impact of commercialisation and genetic data sharing arrangements on public trust and the intention to participate in biobank research. Public Health Genomics.

[50] Mascalzoni D. ELSI of Psychiatrics in Population projects. In: European Biobank Week: 13–16 September 2016; Vienna, Austria. 2016.

[51] Ludman EJ, Fullerton SM, Spangler L, Trinidad SB, Fujii MM (2010). Glad you asked: participants' opinions of re-consent for dbGap data submission. J Empir. Res Hum Res Ethics.

[52] Burstein MD, Robinson JO, Hilsenbeck SG, McGuire AL, Lau CC (2014). Pediatric data sharing in genomic research: attitudes and preferences of parents. Pediatrics.

[53] Trinidad SB, Fullerton SM, Bares JM, Jarvik GP, Larson EB, Burke W (2010). Genomic research and wide data sharing: views of prospective participants. Genet Med.

[54] Budin-Ljosne I, Soye KJ, Tasse AM, Knoppers BM, Harris JR (2013). Genotype-driven recruitment: a strategy whose time has come?. BMC Med Genomics.

[55] 23andMe. https://www.23andme.com/. Accessed 25 Nov 2016.

[56] Biankin AV, Piantadosi S, Hollingsworth SJ (2015). Patient-centric trials for therapeutic development in precision oncology. Nature.

[57] Wynne B (1992). Uncertainty and environmental learning. Glob Environ Chang.

[58] Dove ES, Joly Y, Knoppers BM (2012). Power to the people: a wiki-governance model for biobanks. Genome Biol.

[59] Steinsbekk KS, Kare MB, Solberg B. Broad consent versus dynamic consent in biobank research: Is passive participation an ethical problem? Eur J Hum Genet 2013. 10.1038/ejhg.2012.282 PMC3746258 23299918

[60] Fernandez-Aleman JL, Senor IC, Lozoya PA, Toval A (2013). Security and privacy in electronic health records: a systematic literature review. J Biomed Inform.

[61] Baker DB, Kaye J, Terry SF (2016). Governance Through Privacy, Fairness, and Respect for Individuals. EGEMS (Wash DC).

[62] Beskow LM, Dombeck CB, Thompson CP, Watson-Ormond JK, Weinfurt KP (2015). Informed consent for biobanking: consensus-based guidelines for adequate comprehension. Genet Med.

[63] Sonne SC, Andrews JO, Gentilin SM, Oppenheimer S, Obeid J, Brady K (2013). Development and pilot testing of a video-assisted informed consent process. Contemp Clin Trials.

[64] Genome sequencing: What do patients think? Patient Charter. In: Genetic Alliance UK. 2016. https://www.geneticalliance.org.uk/media/2493/my-cancer-my-dna-patient-charter-edits-sept2016.pdf.Accessed 25 Nov 2016.

[65] Trinidad SB, Fullerton SM, Bares JM, Jarvik GP, Larson EB, Burke W (2012). Informed Consent in Genome-Scale Research: What Do Prospective Participants Think?. AJOB Prim Res.

[66] Tabor HK, Stock J, Brazg T, McMillin MJ, Dent KM, Yu JH (2012). Informed consent for whole genome sequencing: a qualitative analysis of participant expectations and perceptions of risks, benefits, and harms. Am J Med Genet A.

[67] Presidential Commission for the Study of Bioethical Issues: Privacy and Progress in Whole Genome Sequencing. 2012. http://bioethics.gov/sites/default/files/PrivacyProgress508_1.pdf. Accessed 25 Nov 2016.

[68] Precision Medicine Initiative Cohort Program. In: National Institutes of Health. https://www.nih.gov/precision-medicine-initiative-cohort-program. Accessed 25 Nov 2016.

[69] The Precision Medicine Initiative Cohort Program – Building a Research Foundation for 21st Century Medicine - Precision Medicine Initiative (PMI) Working Group Report to the Advisory Committee to the Director, NIH. https://www.nih.gov/precision-medicine-initiative-cohort-program/pmi-working-group. Accessed 25 Nov 2016.

[70] Faglig prioriterte områder i 2016: Interessekonflikter, samtykke og vitenskapelig integritet. In: De nasjonale forskningsetiske komiteene. https://www.etikkom.no/hvem-er-vi-og-hva-gjor-vi/komiteenes-arbeid/faglig-prioritert-omrade-i-2016-interessekonflikter/. In Norwegian. Accessed 25 Nov 2016.

[71] Nishimura A, Carey J, Erwin PJ, Tilburt JC, Murad MH, McCormick JB (2013). Improving understanding in the research informed consent process: a systematic review of 54 interventions tested in randomized control trials. BMC Med Ethics.

[72] Shabani M, Borry P (2015). Challenges of web-based personal genomic data sharing. Life Sci Soc Policy.

[73] Johnsson L, Eriksson S. Autonomy is a Right, Not a Feat: How Theoretical Misconceptions have Muddled the Debate on Dynamic Consent to Biobank Research. Bioethics 2016. 10.1111/bioe.12254 26990222

[74] Mascalzoni D, Hicks A, Pramstaller P, Wjst M (2008). Informed consent in the genomics era. PLoS Med.

[75] McCormack P, Kole A, Gainotti S, Mascalzoni D, Molster C, Lochmuller H, et al. 'You should at least ask'. The expectations, hopes and fears of rare disease patients on large-scale data and biomaterial sharing for genomics research. Eur J Hum Genet. 2016;24(10):1403–8. 10.1038/ejhg.2016.30 PMC5027679 27049302

[76] Lunshof JE, Chadwick R, Vorhaus DB, Church GM (2008). From genetic privacy to open consent. Nat Rev Genet.

[77] Free the data. http://www.free-the-data.org/. Accessed 25 Nov 2016.

